# Analysis of acquisition and retention of cardiopulmonary resuscitation skills according to training frequency

**DOI:** 10.31744/einstein_journal/2025AO1257

**Published:** 2025-10-03

**Authors:** Joyce Kelly Barreto Silva, Thomaz Bittencourt Couto, Andreia Melo Coriolano, Alex Aquino, Júlio Cesar Martins Monte

**Affiliations:** 1 Hospital Israelita Albert Einstein Center for Realistic Simulation São Paulo SP Brazil Center for Realistic Simulation, Hospital Israelita Albert Einstein, São Paulo, SP, Brazil.; 2 Universidade de São Paulo Faculdade de Medicina Center for Medical Education Development São Paulo SP Brazil Center for Medical Education Development, Faculdade de Medicina, Universidade de São Paulo, São Paulo, SP, Brazil.; 3 Hospital Israelita Albert Einstein Faculdade Israelita de Ciências da Saúde Albert Einstein São Paulo SP Brazil Faculdade Israelita de Ciências da Saúde Albert Einstein, Hospital Israelita Albert Einstein, São Paulo, SP, Brazil.

**Keywords:** Cardiopulmonary resuscitation, Simulation training, Clinical skills, High fidelity simulation training, Simultion training, Advanced cardiac life support

## Abstract

**Objective:**

To analyze cardiopulmonary resuscitation skill acquisition and retention at 3 and 6-month intervals and determine the optimal training frequency and associated costs.

**Methods:**

Fifth and sixth-year medical students practiced cardiopulmonary resuscitation using a feedback-equipped simulator.

**Results:**

The study included 43 students. Training with a feedback-equipped device significantly improved the performance compared to baseline: overall performance (median=95% [87–98%] *versus* 60% [18–89%]; p<0.001), compression depth (median=71% [24–92%] *versus* 13% [0–94%]; p<0.001), and compression rate (median=89% [71–98%] *versus* 69% [23–96%]; p=0.002. No significant differences were observed in the total recoil (median=93% [78–99%] *versus* 93% [58–100%]; p=0.991) or hand position (median=99% [100–100%] *versus* 99% [100–100%]; p=0.754). Over time, the overall performance increased by 12% at 3 months (mean ratio [MR]=1.12; p=0.001) and 10.1% at 6 months (MR=1.101; p<0.001). The compression depth improved by 38.9% at 3 months (MR=1.389; p<0.001) and 24.7% at 6 months (MR=1.247; p=0.010), whereas the compression rate increased only at 6 months (MR=1.086; p=0.026). No significant differences were found between the groups trained every 3 months and those trained every 6 months (p>0.05).

**Conclusion:**

Short-term training with a cardiopulmonary resuscitation feedback-equipped simulator significantly improved cardiopulmonary resuscitation skill acquisition and retention. However, no differences were observed between the 3- and 6-month training intervals, suggesting that a 6-month interval may be sufficient for maintaining proficiency.

## Highlights

■All variables that affect the quality of cardiopulmonary resuscitation were analyzed.

■In addition to performance, we analyzed participants’ confidence.

■This study considered the cost of training using the traditional methodology versus feedback.

## In Brief

This study involving 43 fifth- and sixth-year medical students showed that short-term cardiopulmonary resuscitation training using a feedback-equipped simulator significantly improved their performance. The overall performance increased from a median of 60% to 95% (p<0.001) with notable improvements in compression depth (13% to 71%) and rate (69% to 89%). These improvements were retained at both the 3- and 6-month intervals, with no significant difference between them, suggesting that training every 6 months may be sufficient and a cost-effective option.

## Graphical abstract

**Figure:**
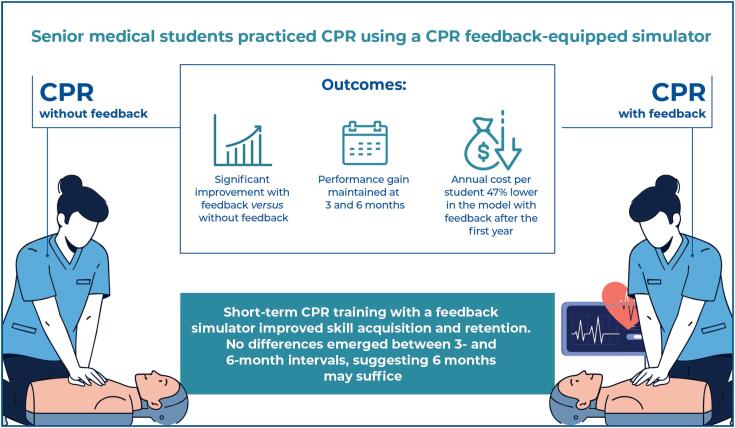

## INTRODUCTION

Various factors can cause cardiorespiratory arrest. Among individuals with coronary artery disease, the annual incidence of cardiac arrest ranges from 20 to 140 per 100,000 individuals. Survival rates remain low globally, ranging from 2% to 11%^( 1 )^ and are below 15% in the United States for both in- and out-of-hospital cases.^( 2 - 4 )^ Despite strong evidence that cardiopulmonary resuscitation (CPR) based on the guidelines from the American Heart Association (AHA) and European Resuscitation Council (ERC) improve the quality of CPR and, consequently, increase survival rates,^( 5 - 11 )^ healthcare professionals still face challenges in delivering high-quality cardiopulmonary resuscitation, often due to inadequate compression depth, rate, chest recoil, or hand positioning.^( 12 - 17 )^ To address this issue, the International Liaison Committee on Resuscitation (ILCOR) introduced the "Formula for survival," which emphasizes the importance of effective CPR training for healthcare providers.^( 18 , 19 )^

Spaced CPR training enhances skill retention and just-in-time training is a feasible option for maintaining competency.^( 20 - 26 )^ However, only few studies have compared the effectiveness of different training frequencies. While some studies support monthly training, others have evaluated intervals of 1, 3, and 6 months. Given the critical role of CPR in patient survival, understanding long-term CPR skill retention is important.

## OBJECTIVE

This study assessed cardiopulmonary resuscitation skill acquisition and retention among medical students trained using a feedback-equipped device at 3- and 6-month intervals.

## METHODS

This randomized controlled trial was conducted at the *Centro de Simulação Realística do Instituto Israelita de Ensino e Pesquisa Albert Einstein (Simulation Center)* and *Faculdade Israelita de Ciências da Saude Albert Einstein (Medical School)* in São Paulo, Brazil.

Initially, all eligible students received a four-part questionnaire sent by email using the REDCap platform. The first part included questions on the eligibility criteria, such as prior CPR training, physical limitations, biological sex, and pregnancy status. To be eligible, students had to be regularly enrolled in the fifth or sixth year of medical school, hold valid Basic Life Support (BLS) and Advanced Cardiovascular Life Support (ACLS) certifications, and have no physical limitations preventing CPR, including pregnancy. The second part contained the informed consent document, and the third part included a sociodemographic questionnaire with questions related to age, height, weight, school year, BLS and ACLS training (in months), experience providing actual or simulated CPR within the past 6 months, and use of feedback-equipped devices in actual or simulated CPR. Finally, the fourth part assessed confidence levels in the domains of overall CPR performance, compression depth, chest recoil, compression rate, and correct hand position ( Supplementary Material -Confidence Questionnaire 1 ).

In the second phase, all participants were invited to participate in individual hands-on CPR training for a maximum of 30 min. First, they watched an instructional video, followed by a checklist to assess their knowledge. Subsequently, the participants completed a baseline practice session using a device without feedback, during which their performance was evaluated for overall quality, compression depth, chest recoil, compression rate, and correct hand position. After initial training, the participants engaged in practice sessions with a feedback-equipped device. Their performance was assessed using the same metrics. The purpose of these assessments was two-fold: to determine baseline CPR performance and to compare subsequent performance with a feedback-equipped device. Then, the participants were randomized into two groups: Group 1 (G1), which underwent CPR training every 3 months, and Group 2 (G2), which underwent training every 6 months.

During CPR training sessions, a simulation technician was responsible for data collection, setting up the simulator, troubleshooting any simulator or equipment issues, and addressing other operational aspects of the study. No facilitators were present during these sessions. The training kit included a CPR cart, simulator with feedback capabilities, computer, step stool, and necessary supplies. Overall performance was analyzed according to the following criteria: the best score out of three attempts, the score of the best attempt in case of interruption due to extreme fatigue, and the score of the best attempt if the performance was deemed "Excellent in the equipament feedback." Participant performance was classified into four levels: basic level – overall performance between 0% and 49%; intermediate level – overall performance between 50% and 74%; advanced level – overall performance between 75% and 100%; and excellent level – overall performance of 90% or above.

The overall performance score was derived from the simulator's feedback screen using the following indicators: compression depth of 50–60 mm, complete chest recoil, compression rate of 100–120/min, and correct hand position. As physicians and medical students at our institution regularly perform CPR in real-life resuscitations using real-time feedback from the Real CPR Helps (ZOLL, Chelmsford, Massachusetts, USA), performance was assessed using the simulator's feedback system.

**Figure 1:**
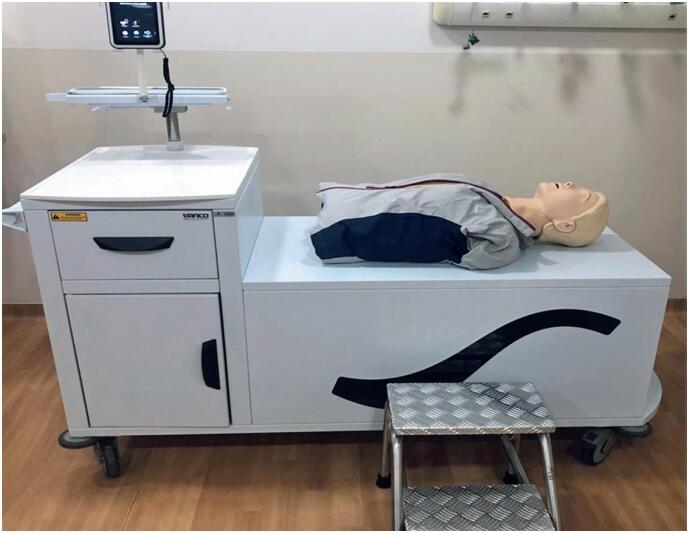
Customized cart with ResusciAnne QCPR Adult simulator (Laerdal Medical, Stavanger, Norway)

The study used the ResusciAnne QCPR Adult simulator (Laerdal Medical, Norway) mounted on a customized cart designed according to the organization's specifications, as shown in figure 1 . This setup enhanced the portability of the device and facilitated implementation of the project in the workplace. The equipment acquired for this study was maintained and operated under the supervision of a simulation technician to ensure optimal functionality.

**Figure 2:**
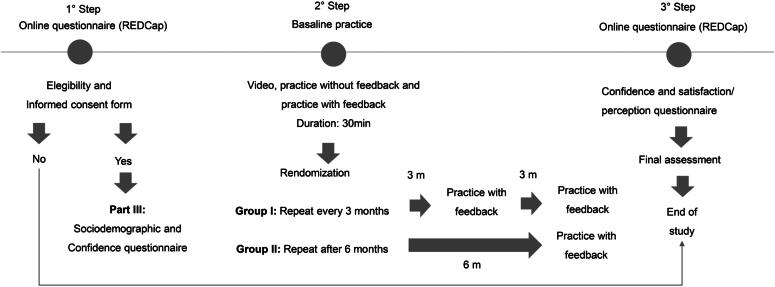
Summary of the methodology

In the third phase, the participants completed a new online form sent by email, which included a confidence level questionnaire identical to the one administered at the onset of the study and a satisfaction survey. The Satisfaction and Perception Questionnaire included questions related to overall satisfaction, contribution of training to professional development, effectiveness of simulation for skill development, adequacy of technological resources, and applicability to in situ simulation. The participants rated their satisfaction or agreement on a scale ( *e.g.* , 1–10 for satisfaction or 1–5 for agreement); ( Supplementary Material -Satisfaction and Perception Questionnaire 2 ). A summary of the methodology used in this study is shown in figure 2 .

Sample size calculation was performed to detect a large effect size between the two groups of interest regarding the rate of excellence, using a χ^2^ test. Assuming a power of 80% and significance level of 5%, the calculated sample size was 39. To account for a potential dropout rate of 30%, 56 participants (28 per group) were included. Calculations were performed using the PWR package. Participants were allocated to groups through randomized block allocation, with the randomization list generated using the blockrand package.

The sample was characterized by mean and standard deviation, minimum and maximum, and median and interquartile range (IQR) for quantitative variables and by absolute and relative frequencies for qualitative variables.^( 27 )^ Data normality was checked using the Shapiro–Wilk test, which is ideal for small sample sizes. In this study, the sizes of the subgroups justified the use of the Shapiro–Wilk test. For larger subgroups, additional methods, such as visual inspections ( *e.g* ., histograms, Q-Q plots), are used to complement the normality assessment.^( 27 )^ Group comparisons were made using the chi-square test or Fisher's exact test, Cochran-Armitage test for qualitative variables, and the Mann–Whitney test for quantitative variables.

To compare performance at the beginning and end of the study, the Marginal Homogeneity Test was used.^( 28 )^ To compare performance throughout the study, generalized estimation equation models were used,^( 29 )^ with the most suitable distribution for the data to include the dependency between more than one measure of the same student. The results are presented as mean ratios and 95% confidence intervals (95%CIs) and p-values. Models with Gamma distribution were used. Analyses were performed using the R package coin and SPPS, v.26.0, with a significance level of 5%.

## RESULTS

Of the 141 students in their fifth and sixth years of medical school, 66 volunteered to participate and met the eligibility criteria. Of them, 62 provided informed consent to participate. Forty-three students were tested at 6 months and included in the final sample. Most of the participants were women (67.4%), and none were pregnant. Most of the participants were in their fifth year of medical school (58.1%). The mean age of participants was 24.7± 2.3 years, mean height was 168.4±8.5cm, and mean weight was 67.53±16.1kg. No significant differences were observed between groups in terms of sex, age, height, or weight. All participants had already undergone BLS (100%), and some had also undergone ACLS (41.9%). The median time since the last BLS was 6 months (range, 2–12), and the median time since ACLS was 3 months (range, 2–3). Only 7% of the participants used feedback-equipped devices in real-life situations, and 76.7% used them in simulated scenarios. Regarding CPR application in the past 6 months, the median was 2.5 (range, 1–5) times in real situations and 3 (range, 2–6) times in simulated scenarios ( Table 1 ).

**Table 1:** Sociodemographic questionnaire

Variable		Total (n=43)	3-month group (n=23)	6-month group (n=20)	p value
Sex, n (%)					
	Female	29 (67.4)	15 (65.2)	14 (70.0)	0.756
	Male	14 (32.6)	8 (34.8)	6 (30.0)	
Age (years)		24.7±2.3	24.7±1.5	24.8±1.6	0.810
Height (cm)		168.4±8.5	168.4±8.9	168.3±9.1	0.965
Weight (kg)		67.53±16.1	67.5±12.3	67.6±11.8	0.978
CPR-related training completed, n (%)					
	BLS	43 (100)	23 (100)	20 (100)	1.000
	ACLS	18 (41.9)	10 (43.5)	8 (40.0)	0.820
Used feedback-equipped devices in real situations?, n (%)					
	No	40 (93.0)	21 (91.3)	19 (95.0)	
	Yes	3 (7.0)	2 (8.7)	1 (5.0)	
Used feedback-equipped devices in simulated situations?, n (%)					
	No	10 (23.3)	5 (21.7)	5 (25.0)	
	Yes	33 (76.7)	18 (78.3)	15 (75.0)	

In the confidence test, significant differences were observed for the following items: "Overall, how confident are you in your CPR skills?" (p=0.007) and "Total chest recoil" (p=0.019). Both items showed a notable increase in confidence levels, with more students rating themselves as "confident" or "fully confident" at 6 months than at baseline. No significant differences were found for "Compression depth between 50–60mm" (p=0.139), "Compression rate between 100–120/min" (p=0.435), or "Correct hand position" (p=0.056). The students’ confidence at the end of the study was compared, and the distribution of responses was similar between the two groups; no significant differences were found in any item (p>0.05). The marginal homogeneity test was used for these comparisons.

**Figure 3:**
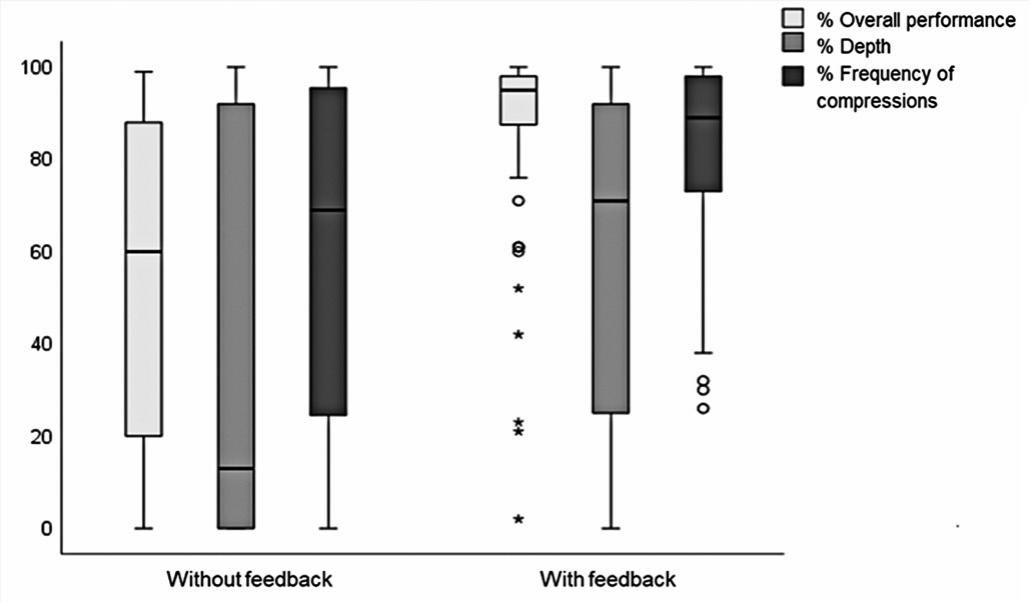
Performance with and without feedback-equipped device (recoil and hand position not shown)

Figure 3 compares student performance levels with and without feedback-equipped device at the first attempt. Significant differences favoring feedback were observed in overall performance (median=95% [IQR: 87–98%] *versus* median=60% [IQR: 18–89%]; p<0.001), compression depth (median=71% [IQR: 24–92%] *versus* median=13% [IQR: 0–94%]; p<0.001), and compression rate (median=89% [IQR: 71–98%] *versus* median=69% [IQR: 23–96%]; p=0.002). No significant differences were found in the total recoil (median=93% [IQR: 78–99%] *versus* median=93% [IQR: 58–100%]; p=0.991) or correct hand position (median=99% [IQR: 100–100%] *versus* median=99% [IQR: 100–100%]; p=0.754). A higher proportion of students achieved "excellent" level with the use of a feedback-equipped device (71.2% *versus* 23.7%; p<0.001).

Table 2 presents a comparison of performance of the 3- and 6-month groups. Data were collected from 43 students: 23 in Group 1 and 20 in Group 2. No significant differences were observed in the performance metrics between the groups (p>0.05). Additionally, the overall performance of Group 1 was compared between the 3- and 6-month assessments, and no significant difference was found (p=0.257).

**Table 2:** Comparison of performance of the 3- and 6-month groups

		3-month group (n=23)	6-month group (n=20)	p value
Overall performance (%)				0.810 ‡
Median and Quartiles		98.0 (96–99)	98.0 (94–99)	
Performance level				0.465 £
	Basic level: 0-49%	0 (0.0)	0 (0.0)	
	Intermediate level: 50-74%	0 (0.0)	1 (5.0)	
	Advanced level: 75-100%	23 (100.0)	19 (95.0)	
Compression depth (%)				>0.999 ‡
	Median and quartiles	77.0 (65–99)	77.5 (59–98)	
Chest recoil (%)				0.634 ‡
	Median and quartiles	93.0 (82–98)	93.5 (81–99.5)	
Compression rate (%)				0.272 ‡
	Median and quartiles	95.0 (87–99)	92.0 (83.5–94.5)	
Correct hand position (%)				0.324 ‡
	Median and Quartiles	100.0 (100–100)	100.0 (99.5–100)	

‡ Mann–Whitney test;

£ Fisher exact test.

We observed an increase in overall performance with the use of a feedback-equipped device of 12% at 3 months (mean ratio [MR]=1.12; p=0.001) and 10.1% at 6 months compared to the baseline performance (MR=1.101; p<0.001). Similarly, we observed an increase in the percentage of compression depth of 38.9% at 3 months (MR=1.389; p<0.001) and 24.7% at 6 months compared to the baseline (MR=1.247; p=0.010). The compression rate showed a significant increase of 8.6% at 6 months compared to the baseline (MR=1.086, p=0.026); however, the increase of 6.6% at 3 months was not significant (MR=1.066, p=0.198). No significant variations were observed in total recoil and correct hand position (p>0.05) at 3 and 6 months compared to the baseline ( Table 1S,Supplementary Material ).

The satisfaction questionnaire responses were compared between the 3-month (n=23) and 6-month groups (n=20). In the 3-month group, 43.5% of students reported being "very satisfied" (score 10), while in the 6-month group, 40.0% gave the same rating. Most students in both groups scored high scores (range, 8–10). Statistical analysis using the Cochran–Armitage test showed no significant differences between the groups (p=0.483). As shown in table 3 , no significant differences were found in students’ perceptions on any of the assessed items (p>0.05). Regarding the frequency of skill refreshers, most students believed that CPR skills should be refreshed every 6 months, with no differences observed between the two groups (p=0.890).

**Table 3:** Perception survey responses

	3 months (n=23)	6 months (n=20)	p value
(1-strongly disagree to 5-strongly agree)			
This activity contributed to my professional development (1-strongly disagree to 5-agree)			0.315 #
4	3 (13.0)	5 (25.0)	
Strongly agree	20 (87.0)	15 (75.0)	
Simulation strategy provides development of CPR skills			0.756 #
4	3 (13.0)	2 (10.0)	
Strongly agree	20 (87.0)	18 (90.0)	
Technological resources were adequate to achieve the proposed goal of learning high quality CPR			0.230 #
4	1 (4.3)	3 (15.0)	
Strongly agree	22 (95.7)	17 (85.0)	
The use of simulation outside the simulation center (at the classroom and during school hours) is an applicable strategy in everyday life			0.379 #
3	0 (0.0)	1 (5.0)	
4	3 (13.0)	3 (15.0)	
Strongly agree	20 (87.0)	16 (80.0)	
How frequently do you feel this skill should be refreshed?			0.890 ‡
Every 3 months	4 (17.4)	2 (10.0)	
Every 6 months	16 (69.6)	15 (75.0)	
Other	3 (13.0)	3 (15.0)	

‡ Fisher exact test;

# Cochran–Armitage test.

CPR: cardiopulmonary resuscitation.

In terms of simulation costs, assuming a batch of 500 students per year, the traditional approach incurs expenses, such as physical room reservations, simulators, medical supplies, and facilitators. In contrast, the feedback-equipped devices allow flexibility as they can be used at existing study, clinical, or common areas. Although facilitators are not required, simulators with feedback require support of a simulation technician. The initial investment in the first year is comparable for both models, with traditional training costing $27 per student and the feedback approach costing $26 per student. However, from the second year onward, the cost of the feedback model decreases to $18 per student. Once the feedback-equipped simulator is established as an asset, the total cost reduces by 47% compared to that of the traditional method ( Table 4 ). The cost savings are likely to continue in subsequent years.

**Table 4:** Annual cost analysis of training with traditional model versus simulator with feedback model

	Traditional Per year	Simulator with feedback	
		1^st^ year	As of 2^nd^ year
Simulation center room, simulator and equipment	$ 8,928	$ -	$ -
Facilitator hours	$ 4,950	$ -	$ -
CPR Simulator with feedback	$ -	$ 4,142	$ -
Technician hours	$ -	$ 8,871	$ 8,871
Total	$ 13,878	$ 13,013	$ 8,871
Cost per student	$ 27	$ 26	$ 18

Data simulated according to the costs of the organization where the study was conducted to train 500 students, based on the year 2021. Prices were converted from Brazilian Reals to US Dollars.

CPR: cardiopulmonary resuscitation.

## DISCUSSION

This study demonstrated a significant improvement in overall CPR performance, including compression depth and rate, when the students used the feedback-equipped device. Participants’ general confidence levels improved over time, and the volunteers expressed satisfaction with the proposed training model. Additionally, long-term skill retention showed an improvement. However, no significant differences in performance were observed between the groups trained at 3- and 6-month intervals. This study focused on the performance of medical school students, a population that has been underrepresented in the existing research on CPR training.

The literature has consistently highlighted the benefits of spaced learning for CPR training; however, the optimal training interval remains unclear. Lin et al. examined the performance of 87 pediatric healthcare providers randomized into a control group with annual BLS training and an intervention group with monthly feedback. They found no differences in the overall CPR performance between the groups, except for improved and sustained pediatric compression depth in the intervention group.^( 26 )^ Similarly, another study involving pediatric healthcare professionals demonstrated that a 3-month high-frequency CPR retraining approach led to improved performance on all variables.^( 30 )^ Another study emphasized the significance of quarterly CPR training and found that improvement was noted among initially poor performers over time, whereas good performers maintained their level of performance. This study strongly supported the use of interval-based learning as a new CPR training method.^( 31 )^

In a study conducted in 2019, quarterly implementation of the Resuscitation Quality Improvement program showed significant improvements in psychomotor skills in CPR within a year. Learners expressed high levels of satisfaction with the training and enhanced confidence in their CPR competency.^( 32 )^ The benefit of retraining is echoed by the consensus regarding the advantages of spaced learning, but only with dissent regarding the optimal frequency of retraining.

Anderson et al. randomly assigned 167 participants to 1-, 3-, 6-, and 12-month training groups. The 1-month training group showed significantly higher percentages of excellent performance (58%) than the 3- (26%), 6- (21%), and 12-month (15%) training groups. Similar to our study, the performances of the 3- and 6-month groups did not differ significantly.^( 12 )^ Oermann et al. also suggested that the dispersion of training sessions from shorter to progressively longer timeframes (beginning with 1–7 days and extending to 30–90 days) could hasten learning curves, reduce variability, and promote maximum performance.^( 33 )^

In our study, baseline CPR performance was inadequate with the traditional model, but it improved significantly with the use of a feedback-equipped device at the beginning of the study, consistent with findings from prior research. However, part of the improvement could be attributed to the consolidation of skills through repetition rather than the use of the feedback-equipped device. Both groups demonstrated improvement in CPR skill acquisition and retention over time. Nevertheless, no significant difference was observed between the groups trained every 3 and 6 months. This result differs from the findings in the literature, which have often demonstrated performance variations based on training frequency. This discrepancy can be attributed to the short observation period, increased volume of real-life consultations during the pandemic, or potential influence of the medical school curriculum. We also observed that participant satisfaction and perceptions were consistently adequate with no significant differences between the groups, although confidence levels increased over time.

A notable aspect of this study is the comparative analysis of the costs of the traditional and feedback-equipped device models. As relevant studies addressing this variable in the given context are scarce, our findings highlight the long-term cost savings and learning benefits associated with the use of the feedback-equipped device, and encourage the use of technological resources.

The sample size may be a limitation of the study as adjustments to the class schedule and clerkship hours due to the COVID-19 pandemic impacted participant recruitment. The small sample size did not allow for a sub-analysis, such as the effect of sex, weight, and height on CPR performance. Another limitation is that fifth- and sixth-year students regularly underwent CPR scenarios, both real and simulated, with instructor feedback, which may have influenced their performance, independent of this study. This is a potential confounding factor, particularly given the unique challenges and adaptations to the pandemic. Further research is needed to address questions related to healthcare professionals and professional categories, which were not included in this study. Additionally, to confirm the actual benefits of feedback-equipped devices, randomization into feedback and no-feedback groups would be a valuable approach for future studies.

## CONCLUSION

Short-term cardiopulmonary resuscitation training using a feedback-equipped simulator significantly improved cardiopulmonary resuscitationskill acquisition and retention. However, no differences were observed between the 3- and 6-month training intervals, suggesting that a 6-month interval may be sufficient to maintain proficiency.

## Supplementary Materials

Analysis of acquisition and retention of cardiopulmonary resuscitation skills according to training frequency

Joyce Kelly Barreto Silva, Thomaz Bittencourt Couto, Andreia Melo Coriolano, Alex Aquino, Júlio Cesar Martins Monte

**DOI: 10.31744/einstein_journal/2025AO1257**

SUPPLEMENTARY MATERIAL 1

**Table 1S:** Comparison of performance assessments

	Mean ratio (95%CI)	p value	Estimated mean (95%CI)
Overall performance (%)			
6 months	1.101 (1.039–1.166)	0.001	94.7 (92.59–96.87)
3 months	1.12 (1.052–1.192)	<0.001	96.38 (94.78–98.02)
Basal (with feedback)	Reference	-	86.05 (80.86–91.57)
Depth (%)			
6 months	1.247 (1.055–1.474)	0.010	74.76 (67.6–82.68)
3 months	1.389 (1.174–1.643)	<0.001	83.24 (76.35–90.75)
Basal (with feedback)	Reference	-	59.95 (51.67–69.56)
Recoil (%)			
6 months	1.02 (0.971–1.072)	0.429	88.43 (85.09–91.91)
3 months	1.013 (0.926–1.109)	0.771	87.84 (80.9–95.38)
Basal (with feedback)	Reference	-	86.68 (82.66–90.89)
Rate (%)			
6 months	1.086 (1.01–1.167)	0.026	88.68 (85.15–92.36)
3 months	1.066 (0.967–1.174)	0.198	87.06 (80.28–94.42)
Basal (with feedback)	Reference	-	81.69 (76.77–86.94)
Hand position (%)			
6 months	1.013 (0.985–1.042)	0.349	99.29 (98.76–99.81)
3 months	1.012 (0.982–1.043)	0.433	99.15 (98.02–100.31)
Basal (with feedback)	Reference	-	97.97 (95.32–100.69)

SUPPLEMENTARY MATERIAL 2

**REDCAP Questionnaire 1:** Confidence questionnaire

**Please indicate how confident you feel in performing the following tasks:**	
**Overall, how confident do you feel in your CPR skills?**	
Achieving a compression depth of 50–60 mm	
	Not Confident
	Slightly Confident
	Neutral
	Confident
	Very Confident
Ensuring full chest recoil	
	Not Confident
	Slightly Confident
	Neutral
	Confident
	Very Confident
Maintaining a compression rate of 100–120/min	
	Not Confident
	Slightly Confident
	Neutral
	Confident
	Very Confident
Correct hand positioning	
	Not Confident
	Slightly Confident
	Neutral
	Confident
	Very Confident

SUPPLEMENTARY MATERIAL 3

**REDCAP Questionnaire 2:** Satisfaction and perception questionnaire

**Thank you for participating in the research study titled "Analysis of Acquisition and Retention of Cardiopulmonary Resuscitation Skills According to Training Frequency."**	
**To complete the data collection process, we kindly ask you to fill out the questionnaire below and the following one.**	
**Please indicate your level of satisfaction regarding the items below:**	
Overall, considering the development of your CPR skills, how satisfied are you?	
	1 (Very Dissatisfied) to 10 (Very Satisfied)
Contribution to your professional development:	
	1 (Strongly Disagree) to 5 (Strongly Agree)
Simulation-based training enables the development of CPR skills:	
	1 (Strongly Disagree) to 5 (Strongly Agree)
The technological resources were adequate to achieve the proposed objectives:	
	1 (Strongly Disagree) to 5 (Strongly Agree)
The use of in situ simulation (at the study location and time) is a strategy applicable to daily practice:	
	1 (Strongly Disagree) to 5 (Strongly Agree)
How often do you believe this skill should be refreshed?	
	Every 3 months
	Every 6 months
	Other
If "Other," please specify:	
______________________________________________________________________________________	

## AVAILABILITY OF DATA AND MATERIALS

Data supporting the findings of this study are available from the corresponding author upon request.
